# Role of the Complement System in the Modulation of T-Cell Responses in Chronic Chagas Disease

**DOI:** 10.3389/fcimb.2022.910854

**Published:** 2022-06-30

**Authors:** María Belén Caputo, Josefina Elias, Gonzalo Cesar, María Gabriela Alvarez, Susana Adriana Laucella, María Cecilia Albareda

**Affiliations:** ^1^ Investigation Department, Instituto Nacional de Parasitología Dr. Fatala Chaben, Buenos Aires, Argentina; ^2^ Chagas Section, Hospital Interzonal General de Agudos Eva Perón, Buenos Aires, Argentina

**Keywords:** *Trypanosoma cruzi*, chagas’ disease, T cell, anaphylatoxins, exhaustion

## Abstract

Chagas disease, caused by the intracellular pathogen *Trypanosoma cruzi*, is the parasitic disease with the greatest impact in Latin America and the most common cause of infectious myocarditis in the world. The immune system plays a central role in the control of *T. cruzi* infection but at the same time needs to be controlled to prevent the development of pathology in the host. It has been shown that persistent infection with *T. cruzi* induces exhaustion of parasite-specific T cell responses in subjects with chronic Chagas disease. The continuous inflammatory reaction due to parasite persistence in the heart also leads to necrosis and fibrosis. The complement system is a key element of the innate immune system, but recent findings have also shown that the interaction between its components and immune cell receptors might modulate several functions of the adaptive immune system. Moreover, the findings that most of immune cells can produce complement proteins and express their receptors have led to the notion that the complement system also has non canonical functions in the T cell. During human infection by *T. cruzi*, complement activation might play a dual role in the acute and chronic phases of Chagas disease; it is initially crucial in controlling parasitemia and might later contributes to the development of symptomatic forms of Chagas disease due to its role in T-cell regulation. Herein, we will discuss the putative role of effector complement molecules on T-cell immune exhaustion during chronic human *T. cruzi* infection.

## Introduction

In an acute infection in which the antigen is totally eliminated, long-lived memory T cells are generated and can be maintained by homeostatic mechanisms regardless of the presence of antigen. In contrast, in the context of chronic infection, the antigen-specific T cells that are generated depend on the presence of the antigen to proliferate, do not respond to homeostatic mechanisms, and lose many of their effector capabilities, raising concerns about the establishment of “true” memory pathogen-specific T cells. This state is called immune exhaustion, and the earliest evidence suggesting the presence of this process was provided by studies using a mouse model of chronic lymphocytic choriomeningitis virus (LCMV) (Gallimore et al., 1998); since then, it has been found to commonly occur during numerous human chronic viral (Boni et al., 2007; Kahan et al., 2015) and bacterial infections (Behar et al., 2014) and, more recently, in protozoan infections, including malaria, *Trypanosoma cruzi* and toxoplasmosis (Gigley et al., 2012; Rodrigues et al., 2014). Immune exhaustion also occurs in noninfectious environments, such as tumors, where tumor antigens are persistently expressed (Ahmadzadeh et al., 2009; Crespo et al., 2013).

Immune exhaustion of T cells is characterized by the progressive and hierarchical loss of effector functions associated with the increased expression of inhibitory receptors, altered gene expression of key transcription factors, cellular metabolic disorders, loss of the homeostatic response to the T-cell cytokines IL-7 and IL-15 and changes in the expression of pro- and anti-apoptotic factors. Finally, the process can culminate with the physical deletion of antigen-specific T lymphocyte clones (Zajac et al., 1998; Gallimore et al., 1998; Doering et al., 2012). Production of cytokine IL-2 and proliferative capacity are the first effector functions to be lost in T cells, while the capacity to produce IFN-γ is the last effector function to be lost (Moskophidis et al., 1993; Oxenius et al., 1998; Welsh, 2001; Wherry et al., 2003). However, exhausted T cells are still important for controlling the host–pathogen equilibrium in a chronic infection (Schmitz et al., 1999). In addition to the T-cell compartment, the innate immune system is affected during chronic infections (Zuniga et al., 2015; Greene and Zuniga, 2021). The complement system, which is a key effector of innate immune responses in acute infections, has also been shown to be involved in the modulation of adaptive immune responses (Ricklin et al., 2010).

The immune system is central to the control of *Trypanosoma cruzi* infection; when it is not sufficient, the parasite load and inflammation, and therefore, the potential for tissue damage, increases (Tarleton et al., 1994). Our group has shown that persistent infection by *T. cruzi* induces exhaustion of *T. cruzi*-specific T-cell responses, which is also reflected by a high degree of differentiation and activation of T cells in the circulation of subjects chronically infected with the parasite (Laucella et al., 2004; Albareda et al., 2006; Alvarez et al., 2008; Albareda et al., 2009; Albareda et al., 2013; Albareda et al., 2015).

In recent decades, much knowledge has been gained about the complexity of the complement system, which has more than just a defense role in the circulation during acute infections and is related to complement-mediated intracellular and autocrine regulation involved in the modulation of the innate and adaptive immune responses. Increased levels of complement anaphylatoxins in subjects with chronic *T. cruzi* infection have been reported (Ndao et al., 2010) but whether the complement system has an immunoregulatory role in T-cell exhaustion during chronic Chagas disease remains unknown. Understanding the interaction between complement molecules and the T-cell response is important in the setting of a chronic infection. In this minireview, we will discuss the putative role of effector complement molecules on the T-cell immune exhaustion during chronic human *T. cruzi* infection.

## Development of T-Cell Responses and Immune Exhaustion in Chronic Human *Trypanosoma Cruzi* Infection

Chagas disease, caused by the intracellular pathogen *Trypanosoma cruzi*, is the parasitic disease in Latin America with the highest impact and is the most common cause of infectious myocarditis in the world (WHO, 2018). The infection is naturally transmitted by blood-feeding Reduviid insects, but transmission by oral contamination, transplacental transmission and transmission by blood transfusion/tissue transplantation are also possible. The main reason that Chagas disease has been described as having an autoimmune etiology is that detection of the parasites is difficult, but there is an emerging consensus that the persistence of parasites is the primary cause of cumulative tissue damage in chronic Chagas disease (Tarleton, 2003). Since *T. cruzi* presents two different anatomical development stages in the mammalian host, trypomastigotes in the bloodstream and amastigotes in the cytoplasm of the infected cells, immune control of the parasite requires the generation of both innate and adaptive immune responses. However, even in cases in which those immune responses are sufficient to control the acute infection, the parasite is not completely cleared, resulting in a decade-long infection in most cases (Tarleton, 2007). The persistence of these inflammatory responses may eventually result in the tissue damage that is found in subjects chronically infected with *T. cruzi* (Tarleton, 2001). These data also support the hypothesis that stimulation of an effective set of immune responses that efficiently limit the parasite load in tissues should result in less severe disease.

One of the reasons why the inflammatory process might be exacerbated in the more severe forms of the disease is that *T. cruzi*-specific T-cell responses become less effective due to the process of immune exhaustion (Laucella et al., 2004; Albareda et al., 2006; Albareda et al., 2015; Lasso et al., 2015; Egui et al., 2015; Ferreira et al., 2017). It has been shown that cells producing only one cytokine (i.e., monofunctional T cells) in response to *T. cruzi* antigens is common in adults with chronic Chagas disease (Laucella et al., 2004; Lasso et al., 2015; Mateus et al., 2015), while polyfunctional T cells are often found in children who have shorter-term infections (Albareda et al., 2013). We have demonstrated that the impairment of parasite-specific T-cell responses is inversely correlated with the severity of the disease (Laucella et al., 2004; Albareda et al., 2006; Alvarez et al., 2008) and increases with the length of the infection (Albareda et al., 2013).

We have shown that higher frequencies of IFN-γ-producing T cells specific for *T. cruzi* are detected in subjects chronically infected with *T. cruzi* without or with mild clinical symptoms compared to subjects in the more severe stages of the disease. On the other hand, IL-2-secreting T cells are infrequent in chronic infections regardless of clinical status (Laucella et al., 2004; Albareda et al., 2006; Alvarez et al., 2008). This functional profile of IFN-γ-only secreting T cells, characteristic of effector memory T cells and generally associated with long-term antigen persistence and exhausted T cells (Harari et al., 2006), is consistent with other features of exhausted T cells found in subjects with long-term *T. cruzi* infections, such as the increased frequency of fully differentiated memory T cells, a high rate of apoptosis, high expression of inhibitory receptors, alterations in IL7/IL7R axis and high dependence on the presence of antigen for T-cell maintenance (Albareda et al., 2006; Laucella et al., 2009; Argüello et al., 2012; Lasso et al., 2015; Albareda et al., 2015; Natale et al., 2018). It is important to note that even though these T cells might be effective in controlling the infection in subjects chronically infected with *T. cruzi*, as long as the efficiency of the immune response declines, the ability to control the parasite burden without increasing the level of tissue damage appears to be diminished.

## Functions of the Complement System

Regardless of the pathway used (classical, alternative and lectin pathways) the activation of the complement system results in the generation of the C3 and C5 convertases which are cleaved into the main effectors molecules, the C3a and C5a anaphylatoxins and the opsonins C3b and C5b. The C3a and C5a molecules induce pleiotropic effector functions by binding to their specific receptors C3aR and C5aR1/2, respectively (Klos et al., 2009), C5b leads to the formation of the membrane attack component (MAC) and C3b is related to the phagocytosis of the opsonized targets (Ricklin et al., 2010), The balance between the activation and inhibition of the complement system is essential to protect cells from damage induced by an indiscriminate level of immune response. Complement activation is finely regulated by complement regulatory proteins (Cregs), including soluble (C1 inhibitor, factor H, and C4b-binding protein) and membrane-associated (CD46, CD55, CR1, and CD59) proteins, which regulate the activation of the complement cascade, mainly at the C3 convertase stage (Kim and Song, 2006; Zipfel and Skerka, 2009; Ricklin et al., 2016; Arbore et al., 2017).

The complement system is usually thought of as a serum circulating and membrane-bound protein system where the liver is the main source of their components and the main function is the detection and elimination of circulating pathogens as part of the innate immune response (Ricklin et al., 2010). However, the findings that most of immune cells can produce complement proteins and express their receptors have led to the notion that the complement system also has non canonical functions in the adaptive immune response (Klos et al., 2009; Ricklin et al., 2010; Killick et al., 2018). This intracellular complement, was named as complosome to set it apart from the liver-derived and serum-circulating complement system (Killick et al., 2018). Complement molecules might act indirectly or directly on T cells, either promoting or inhibiting immune responses (Heeger and Kemper, 2012; Clarke and Tenner, 2014; Liszewski et al., 2017; Moro-García et al., 2018; West et al., 2018).

**Figure 1:**
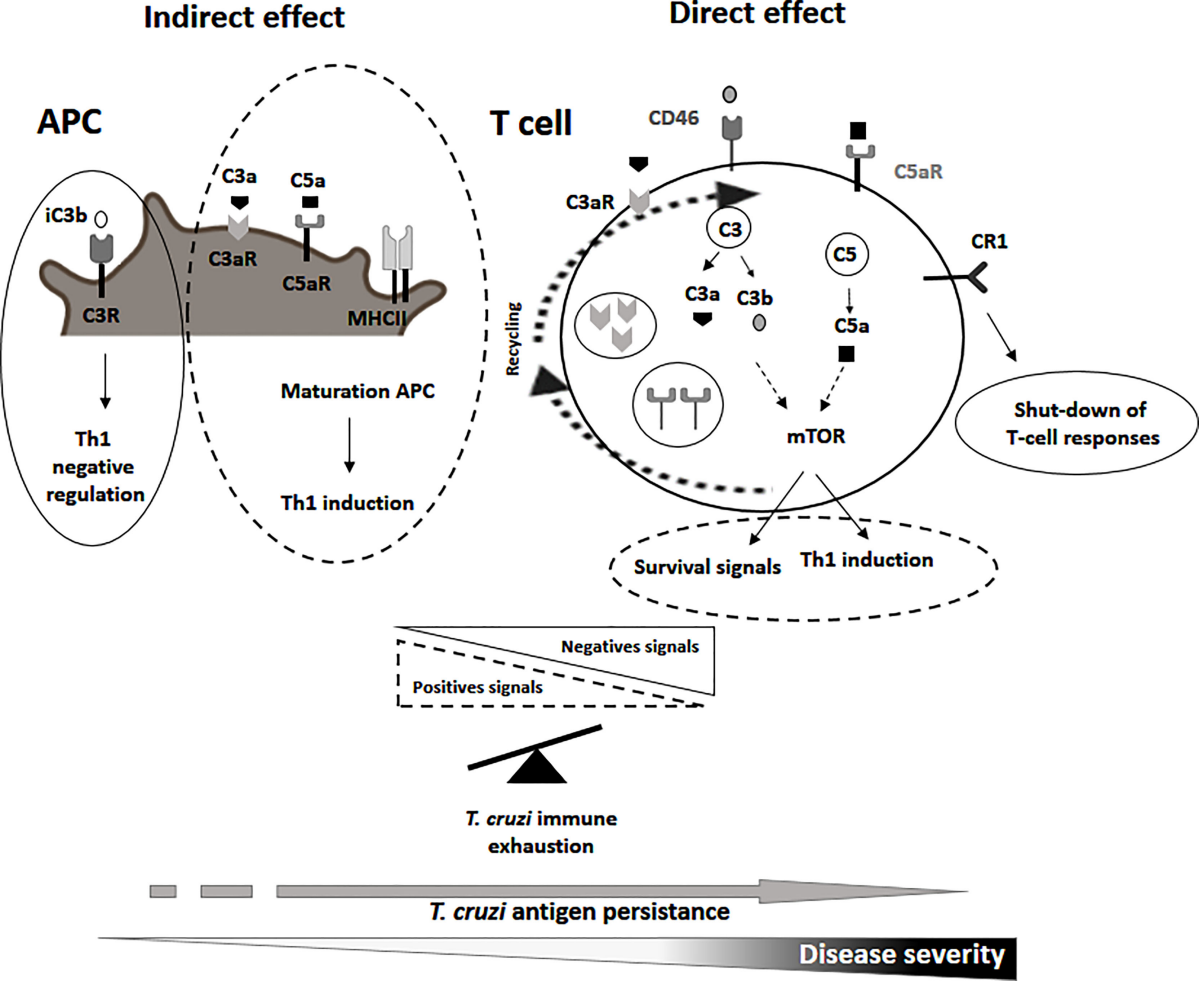
Representative scheme of the non canonical functions of the complement molecules in the putative roles on T-cell immune exhaustion during chronic human *T. cruzi* infection. Possible roles of the C3a, C3b, C5a, their receptors and complement receptor 1 and CD46 in T-cell exhaustion process are indicated. Dashed lines indicate positive signals involved in the maturation of APC, induction of a Th1 response or T-cell survival. Complete lines indicate negative signals involved in the control of a Th1 response.

Regarding the indirect effects, signals delivery after the activation of the C3aR, C5aR by the binding of C3a and C5a, respectively, induce the maturation of the APC (i.e. dendritic, macrophages, and monocytes) by the secretion of interleukin IL-12, upregulation of the major histocompatibility class II necessary for the induction of a good Th1 response (Kemper and Atkinson, 2007). In contrast, the binding of the C3R by the inactivated form C3b (iC3b) on the APCs activates the production of the regulatory cytokines IL-10 and TGF-b which act as a negative regulators of T-cell responses (**Figure 1**; Sohn et al., 2003). In relation to the direct effects of the complement system on the T-cell function, following antigen stimulation C3a and C3b are translocated to the T-cell membrane where they engage their receptors, C3aR and the costimulatory CD46 (West et al., 2018), respectively. These interactions mediates the cellular metabolic reprogramming of T cells by the induction of the mTOR program involved in the upregulation of the aerobic glycolysis and oxidative phosphorylation process necessary for the induction of the Th1 response (Figue 1; Liszewski et al., 2017; West et al., 2018). Additionally, there is downregulation of the membrane-bound complement negative regulator CD55 further promoting the differentiation into Th1 effector cells (Lalli et al., 2007). On the other hand, the activation of the complement receptor 1 (CR1) in T cells might inhibit IL-2 production, and proliferation, and promote the secretion of IL-10 leading to a shut-down of the T-cell response (**Figure 1**).

The complement system is also involved in T-cell homeostasis through the binding of C3b to CD46 promoting the expression of the IL7R (CD127) (Wofford et al., 2008; Carrette and Surh, 2012; Kolev et al., 2014; Clarke and Tenner, 2014). There is a basal metabolic activity of the intracellular C3a/C5a and receptor recycling pathway that promote T-cell survival *via* a low level activation of mTOR (**Figure 1**; West and Kemper, 2019).

In summary, these data support the interplay between complement effector molecules and their receptors on APCs and T cells which might influence the outcome of T-cell responses in the context of chronic Chagas disease and the mechanism of T-cell exhaustion.

## Possible Roles of the Complement System in T-Cell Exhaustion in Chronic Chagas Disease

The majority of people infected with *T. cruzi* remain asymptomatic throughout their lives, and parasite-host interactions seem to play a crucial role in the development of the disease. It is thought that inflammation in the heart develops over years from indolent, low-grade processes that depend, at least in part, on the few parasites that persist in the heart (Bonney et al., 2019), inducing constant activation of the immune system. Few studies have addressed the role of the complement system in the chronic phase of *T. cruzi* infection. A study by Aiello et al. (2002) using frozen myocardial samples from chronic Chagas disease patients with cardiomyopathy found that the presence of *T. cruzi* parasites in heart necropsies was associated with increased complement activation and MAC deposition

Thus, the release of effector complement molecules with canonical functions might contribute to the development of symptomatic forms of chronic infection due to their proinflammatory effect and also impact in the continuous inflammatory reaction due to parasite persistence in the heart that leads to necrosis and fibrosis in subjects chronically infected with the parasite. (Boldt et al., 2011; Weitzel et al., 2012; Luz et al., 2016). In contrast, other studies have described increased levels of C3a in the sera of subjects with asymptomatic chronic Chagas disease supporting a positive role of the complement system in chronic *T. cruzi* infection (Ndao et al., 2010).

An exacerbation of the negative regulatory mechanisms or inappropriate activation of positive signals induced by the non-canonical functions of the complement system might participate in the process of T-cell exhaustion in chronic Chagas disease by a shut-down of *T. cruzi*-specific T-cell responses. A deficiency in the maturation of the APC, a low activation of the mTOR pathway or the CD46 signaling by a deficient expression of C3aR, C5aR,C3a, C3b and C5a, might induce a poor Th1 response. An imbalance of complement-mediated negative over positive signals would further inhibit T-cell responses (**Figure 1**). Homeostatic T-cell survival might be also compromised by a downregulation of C3aR, C5aR, C3a, C3b and C5a (Le Friec et al., 2012; Clarke and Tenner, 2014).

A better understanding of the regulatory mechanisms of the complement system in T-cell responses might provide new insight in this chronic parasitic disease.

## Author Contributions

All authors listed have contributed in the preparation and organization of the work, and approved it for publication. All authors contributed to the article and approved the submitted version.

## Funding

This work was supported by the Scientific and Technological Research Fund (FONCyT), Argentina (PICT 2019-00157 to MCA). MCA and SLA are members of the Scientific Career, CONICET, Argentina. MBC and GC are CONICET Ph.D. fellows. JE is a fellow of the Scientific and Technological Research Fund (FONCyT), Argentina.

## Conflict of Interest

The authors declare that the research was conducted in the absence of any commercial or financial relationships that could be construed as a potential conflict of interest.

## Publisher’s Note

All claims expressed in this article are solely those of the authors and do not necessarily represent those of their affiliated organizations, or those of the publisher, the editors and the reviewers. Any product that may be evaluated in this article, or claim that may be made by its manufacturer, is not guaranteed or endorsed by the publisher.
